# Retroperitoneal Mature Cystic Teratoma in a Neonate: A Case Report

**Published:** 2016-04-10

**Authors:** Prashant Sadashiv Patil, Paras Kothari, Abhaya Gupta, Vishesh Dikshit, Ravi Kamble, Geeta Kekre, Shahaji Deshmukh

**Affiliations:** Department of Pediatric Surgery, Lokmanya Tilak Municipal Medical College and General Hospital, Mumbai, India

**Keywords:** Mature teratoma, Neonate, Retroperitoneum

## Abstract

We report a case of retroperitoneal mature cystic teratoma in a 2-day-old neonate. Diagnostic and surgical procedure including its complexity and relevant literature review has been discussed.

## CASE REPORT

A 2-day-old female neonate, weighing 2.7 kg, antenatally diagnosed at 30 weeks of gestation as a case of abdominal mass with polyhydramnios in primipara mother referred to our department for further management. Baby was delivered normally. Abdominal examination revealed a lump in left side of abdomen extending from left hypochondriac region to left iliac fossa and crossing midline in umbilical region. Lump had variegated consistency, nodular surface, clearly defined margins and restricted mobility. Abdominal X-ray showed bowel loops pushed to right side of abdomen by a space occupying lesion in left side with foci of calcifications in it. Post natal abdominal ultrasound revealed a large heterogeneous solid cystic lesion with few echogenic areas and calcifications. Computed tomography scan of abdomen revealed 10.8 x 8.3 x 8.4 cm heterogeneously enhancing cystic/solid mass lesion with multiple foci of discrete calcifications in retroperitoneum (Fig. 1). Lesion was partially encasing aorta, and displaced major vessels around. Serum alpha-fetoprotein (AFP) level was 28675 IU/ml. 

**Figure:**
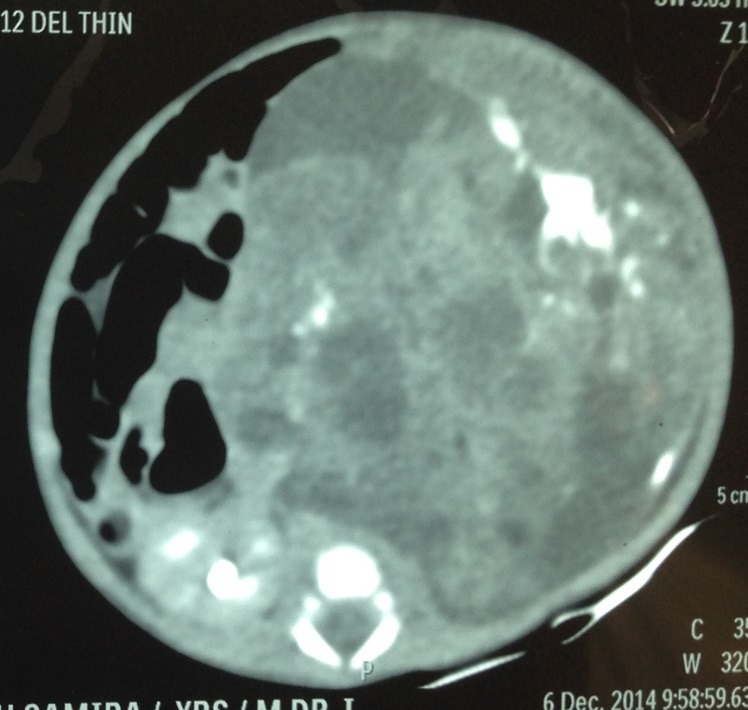
Figure 1: CT scan of the abdomen showing solid cystic tumor in retroperitoneum with foci of calcification

Tru-cut biopsy was taken under anesthesia which came out as mature teratoma. Baby underwent laparotomy at day 24 of life. A large retroperitoneal tumor (12 x 8 cm) consisting of solid and cystic areas occupying mainly left flank and central abdomen. The tumor was adherent to the surrounding structures namely the root of mesentery, superior mesenteric vessels, inferior mesenteric vessel, duodenum, colon, and left kidney and renal vessels. Tumor was displacing aorta and inferior vena cava to right side. However, there was no infiltration into any vessels or organs neither was any significant lymphadenopathy noted. Left kidney was found to be pushed down into pelvis by tumor. Tumor (800 gm) was excised in toto (Fig. 2). 

**Figure:**
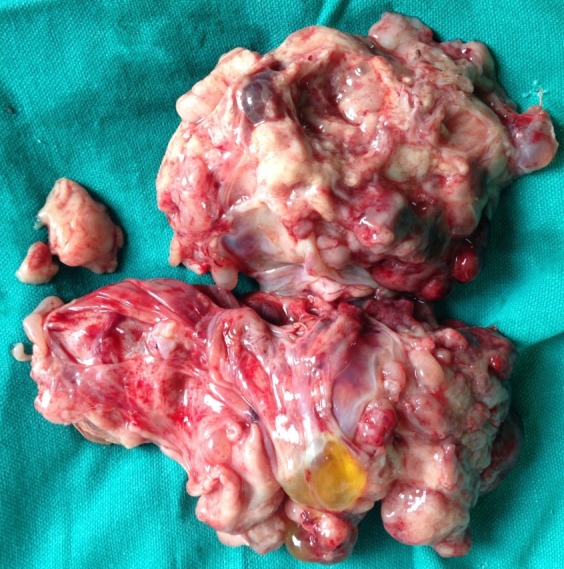
Figure 2: Cut surface of excised tumor

Post-operatively, the patient was kept on ventilator and extubated on 2nd post-op day. Baby was discharged on 11th post-op day on full oral feeds and followed up monthly. Serum AFP at 2 months and 6 months were normal. Baby has gained adequate weight and doing well. Histopathological examination of tumor revealed presence of neural tissue, spindle cells, cartilage, fibroblasts, hypoplastic bone.

## DISCUSSION

Teratoma is the commonest congenital tumor. The usual site for congenital teratoma is sacrococcygeal, the other sites being the mediastinum, head and neck, oropharynx, pericardium, and the retroperitoneum [1-3]. Retroperitoneal teratoma comprises 3.5-4% of all germ cell tumors in children and 1-11% of primary retroperitoneal neoplasms [4-6]. Patients usually present with abdominal distension or a palpable mass. An accurate diagnosis of a teratoma cannot be made on clinical basis. Radiological features include presence of calcification, teeth and fat, however calcification cannot be considered an indicator of a benign tumor since 12.5% of calcified tumor are malignant. Ultrasound of abdomen is usually the first imaging modality used in pediatric abdominal mass. CT scan is useful to delineate the extent of the disease in retro-peritoneum and its relationship to major vessels. Serum alpha-feto protein level is good indicator for diagnosis and assessing the recurrence of tumor. Malignancy is uncommon in retro-peritoneal teratoma hence non mutilating excision is possible and should be attempted [7, 8]. The prognosis of neonatal teratoma is favorable with an 80-100% survival reported after surgical excision of the tumor and treatment of any recurrence [5, 6]. In our case, the patient was a neonate and retroperitoneal teratoma is very unusual in this age group and at this site. Postoperative recovery was uneventful. At present there is no sign of recurrence of malignancy at follow up of 10 months. 

To conclude, retroperitoneal teratoma can present in neonates however early diagnosis and management like done in the index case can give good outcome.

## Footnotes

**Source of Support:** Nil

**Conflict of Interest:** Nil
